# Cocaine in Surgery

**Published:** 1893-08

**Authors:** 


### Cocaine in Surgery.

Reclus, in *La Semaine Medicale*, thinks cocaine is a most valuable agent under certain conditions, as it enables delicate and important operations to be performed without assistance, and its use is not attended with the same risk as chloroform. He has used it for amputation of the forearm, in the case of an old man of 83, who was worn out with prolonged suppuration. The solution was injected into the various tissues as they were met with. He thinks that stronger solutions than one or two per cent should not be used, and that the dose of cocaine injected should not exceed from two to three grains. The patient should be recumbent. The fluid should be introduced gradually, and from three to six minutes allowed, according to the strength of the solution, before the incision is made

